# An Improved System for Generation of Diploid Cloned Porcine Embryos Using Induced Pluripotent Stem Cells Synchronized to Metaphase

**DOI:** 10.1371/journal.pone.0160289

**Published:** 2016-07-29

**Authors:** Eunhye Kim, Zhong Zheng, Yubyeol Jeon, Yong-Xun Jin, Seon-Ung Hwang, Lian Cai, Chang-Kyu Lee, Nam-Hyung Kim, Sang-Hwan Hyun

**Affiliations:** 1 Laboratory of Veterinary Embryology and Biotechnology, (VETEMBIO), Veterinary Medical Center and Collage of Veterinary Medicine, Chungbuk National University, Cheongju, Chungbuk, Republic of Korea; 2 Institute for Stem Cell & Regenerative Medicine (ISCRM), Chungbuk National University, Cheongju, Chungbuk, Republic of Korea; 3 Department of Animal Sciences, Agriculture, Life, & Environmental Sciences, Chungbuk National University, Cheongju, Chungbuk, Republic of Korea; 4 Department of Agricultural Biotechnology, Research Institute for Agriculture and Life Science, Seoul National University, Seoul, Republic of Korea; Friedrich-Loeffler-Institute, GERMANY

## Abstract

Pigs provide outstanding models of human genetic diseases due to their striking similarities with human anatomy, physiology and genetics. Although transgenic pigs have been produced using genetically modified somatic cells and nuclear transfer (SCNT), the cloning efficiency was extremely low. Here, we report an improved method to produce diploid cloned embryos from porcine induced pluripotent stem cells (piPSCs), which were synchronized to the G2/M stage using a double blocking method with aphidicolin and nocodazole. The efficiency of this synchronization method on our piPSC lines was first tested. Then, we modified our traditional SCNT protocol to find a workable protocol. In particular, the removal of a 6DMAP treatment post-activation enhanced the extrusion rate of pseudo-second-polar bodies (p2PB) (81.3% vs. 15.8%, based on peak time, 4hpa). Moreover, an immediate activation method yielded significantly more blastocysts than delayed activation (31.3% vs. 16.0%, based on fused embryos). The immunofluorescent results confirmed the effect of the 6DMAP treatment removal, showing remarkable p2PB extrusion during a series of nuclear transfer procedures. The reconstructed embryos from metaphase piPSCs with our modified protocol demonstrated normal morphology at 2-cell, 4-cell and blastocyst stages and a high rate of normal karyotype. This study demonstrated a new and efficient way to produce viable cloned embryos from piPSCs when synchronized to the G2/M phase of the cell cycle, which may lead to opportunities to produce cloned pigs from piPSCs more efficiently.

## Introduction

Pigs are regarded as a powerful pre-clinical research tool because of their appropriate organ size, lifespan and similar anatomical and physiological attributes in comparison with humans, along with greater ease of use and availability compared to non-human primate models [1, 2]. Furthermore, genetically modified pigs have many potential applications in agricultural and biomedical research [3]. The recent development of nucleases (CRISPR, TALENs and ZFN technologies) have enabled the highly efficient generation of knockout animals and a revolution in animal transgenesis [4–6]. Porcine genetic engineering has been hampered because there have been no suitable embryonic stem cell (ESC) lines capable of germ cell contribution [7]. Instead, genetically modified pigs have been produced using genetically modified somatic cells and nuclear transfer (NT) [8]. However, current somatic cell nuclear transfer (SCNT) efficiencies using pigs are relatively low, with only 1–3% developing to term without showing abnormalities at birth until now.

One of the important aspects regarding this problem is associated with the synchronization of donor nuclei and the recipient oocyte [9–11]. Although the optimal cell cycle of donor cells is still controversial, the donor nuclei are usually required to be arrested at the G0/G1 or early S phase of the cell cycle for the maintenance of normal ploidy in the reconstructed NT embryo [12]. In this context, the first cloned pigs were generated from somatic cells induced into quiescence by serum starvation [13]. However, extended serum starvation of more than 2 days does not further enhance the proportion of cells at the G0/G1 stage [14], but instead reduces cell survival, leading to more DNA fragmentation and embryonic losses [15, 16]. In contrast, Lai et al. demonstrated that the G2/M-stage synchronized nuclei of fetal fibroblast donors can be morphologically remodeled by the cytoplasm of MII oocytes in pig and have shown feasibility in producing NT embryos, although they still had problems associated with lowered normal ploidy rates [17].

Previous works have reported that another major problem in nuclear cloning is related to incomplete epigenetic reprogramming of the donor nucleus, resulting in aberrant gene expression during development [18–21]. Therefore, the use of small molecular reprogramming modifiers such as histone deacetylase inhibitors (HDACi) has been recently attempted to improve cloning efficiency [22]. In this regard, there is evidence to propose that undifferentiated cells can improve the efficiency of NT because they exhibit enhanced proliferative capacity and are more easily reprogrammed than differentiated somatic cells [23]. For example, clones reconstructed with ESCs have been shown to result in more efficient cloning and fewer abnormalities in offspring born, compared with those reconstructed with adult cells.

Induced pluripotent stem cells (iPSCs) have been generated by reprogramming somatic cells from multiple mammalian species using defined cocktails of transcription factors [24]. Viable mice have been produced from mouse iPSCs through tetraploid complementation, as well as NT [25–27], which show that iPSCs are very similar to ESCs and could serve as a substitute for ESCs for the purpose of genomic manipulation. The generation of porcine iPSCs (piPSCs) have also been reported by several groups [28–30]. Because piPSCs are capable of long-term proliferation, they may allow for lengthier *in vitro* manipulations. Moreover, they are similar to ESCs in many aspects, suggesting that they may also have highly efficient homologous recombination capabilities. Thus, the attempt to produce cloned pigs from piPSCs was performed by various research groups, but yielded almost complete failures, even when using cell cycle controlled donors [31–33]. The cloning efficiency was extremely low and the only viable piglets were produced by hand-made cloning but not the common NT method. The low efficiency may be due to the status of donor cells, resulting in low blastocyst formation rates and, more importantly, mosaic or aneuploid blastocysts.

The aim of the present study was to produce diploid cloned porcine embryos from piPSCs more efficiently. To do this, we adopted a double-blocking method to synchronize our piPSCs cells to metaphase, then used them as donor cells for NT. We observed the pseudo-second-polar body (p2PB) extrusion, compared the preimplantation developmental competence of piPSCs nuclear transfer (piPSNT) embryos produced by different protocols and checked the genomic normality of these embryos.

## Materials and Methods

### Ethics Statement

This study was carried out in strict accordance with the recommendations in the Guide for the Care and Use of Laboratory Animals of the National Veterinary and Quarantine Service. The protocol was approved by the Committee on the Ethics of Animal Experiments of the Chungbuk National University (Permit Number: CBNUA-584-13-01). All sacrifice was performed under isoflurane anesthesia, and all efforts were made to minimize suffering.

### Chemicals

All chemicals and reagents used in this study were purchased from Sigma-Aldrich Chemical Company (St. Louis, MO, USA), unless otherwise stated.

### Preparation of mouse embryonic fibroblasts (MEFs) as the feeder cell layer

The feeder cell layer was prepared using embryonic day 13.5 ICR mouse fetuses. The ICR mice were purchased from Daehan Biolink (Chungnam, Republic of Korea). Fetal heads, internal organs and legs were removed, after which the remaining tissues were minced in PBS and centrifuged at 2000 rpm for 3 min at least twice until MEFs were obtained. MEFs were cultured in Dulbecco’s modified Eagle’s medium (DMEM; Gibco, Carlsbad, CA, USA) with 10% FBS (Gibco, Carlsbad, CA, USA), 1% non-essential amino acids (Gibco, Carlsbad, CA, USA), 1% glutamine (Gibco, Carlsbad, CA, USA), 0.1 mM ß-mercaptoethanol (Gibco, Carlsbad, CA, USA) and 1% antibiotic-antimycotic (Gibco, Carlsbad, CA, USA) (growth medium) at 37°C under 5% CO2 in air. To inactivate MEFs, they were treated with 10 μg/mL mitomycin C (Roche, Basel, Switzerland) at passage 2–3 for 2–2.5 h prior to their use as cultured pig blastocysts. Inactivated MEFs were plated at a density of 2.5 x 105 cells/mL in a 4-well dish coated with 0.5% gelatin in growth medium.

### Porcine induced pluripotent stem cell culture and cell cycle synchronization

The piPSCs (piPSN-1) were kindly provided by professor Chang-Kyu Lee from Seoul National University. The piPSN-1 lines were produced by transfecting porcine embryonic fibroblasts (PEFs) with four doxycycline inducible human factors (FUW-tetO-hOct4, FUW-tetO-hSox2, FUW-tetO-hKlf4, FUW-tetO-hc-Myc and FUW-M2rtTA from Addgene) by lentivirus as previously described [28, 34].

The cells were cultured in porcine ESC medium (50:50 mixture of DMEM and F10, supplemented with 15% FBS, 2 mM glutamax, 0.1 mM ß-mercaptoethanol, 1x MEM nonessential amino acids, 1x antibiotic/antimitotic) supplemented with 1000 units/ml LIF (LIF2010, Millipore), 2 μg/ml doxycycline (D9891, Sigma), 1 μM PD0325901 (MEK inhibitor, Stemgent) and 3 μM CHIR99021 (GSK3 inhibitor, Stemgent) on mitotically inactivated MEFs in 4-well dishes. Cells were cultured at 38.5°C in a 5% O2 and 95% air atmosphere. The medium was changed daily and the cells were passaged every two or three days. These cells showed mouse ESC-like morphologies and expressed Oct4, Sox2, Nanog, SSEA1 and SSEA4. They could be passaged using a 0.04% trypsin treatment and maintained a stable morphology for more than 50 passages [35]. The piPSC lines used in this study displayed mouse ESC-like colony morphology and maintained stable proliferation capabilities. They expressed pluripotent markers such as OCT4, SOX2, NANOG, SSEA1 and SSEA4 in the present of doxycycline, LIF and a two inhibitor (2i) formulation comprised of the MEK inhibitor with the GSK3 inhibitor. Cells between passage 30 and 40 were used in the following experiments.

Synchronization of piPSCs was conducted using a double blocking method as previously reported [33]. Briefly, 0.3 μM aphidicolin (A0781, Sigma) was added to the above mentioned cell culture medium for 10 h to arrest the cells in S phase. After release from aphidicolin treatment for 1 h, the cells were treated with 20 ng/ml nocodazole (M1404, Sigma) for another 4 h. Then, the cells were harvested and suspended in nocodazole-supplemented culture medium until use.

### *In vitro* maturation of porcine oocytes

Ovaries were collected from prepubescent gilts at a local slaughterhouse and transported to the laboratory within 1 h in saline at 37°C. We received permission to use the ovaries from Chungbuk Veterinary Service. Porcine ovaries were provided by the regional slaughterhouse (HanNaeng, Cheongwon, Republic of Korea). Cumulus-oocyte complexes (COCs) were aspirated from follicles at 3–6 mm in diameter using 18-gauge needle attached to a 10-ml syringe. Oocytes possessing an evenly granulated cytoplasm and a compact surrounding cumulus mass were collected and washed twice with TL–HEPES–PVA medium (Tyrode’s lactate–HEPES medium supplemented with 0.01% polyvinyl alcohol). After washing, 50–60 COCs were transferred to 500 μl of in vitro maturation (IVM) medium (TCM-199, Invitrogen, Carlsbad, CA) supplemented with 10 ng/ml epidermal growth factor (EGF), 1 mg/ml insulin (Sigma-Aldrich, St. Louis, MO), 4 IU/ml eCG (Intervet, Boxmeer, The Netherlands), hCG (Intervet) and 10% (v/v) porcine follicular fluid. After 22 h of culture, the COCs were transferred to hormone-free IVM medium and cultured for additional 18, 22 or 26 h at 38.5°C under 5% CO2 in humidified air. Denuded oocytes with obvious first polar bodies and uniform ooplasm were selected for the nuclear transfer experiments.

### Nuclear transfer and oocyte activation

Oocytes were first incubated in manipulation medium (TLH-BSA, calcium-free TLH containing 0.2% bovine serum albumin) containing 5 μg/ml cytochalasin B (C6762) and 5 μg/ml Hoechst 33342 (B2261, Sigma) for 5 min. After washing several times in TLH-BSA, the oocytes were enucleated by aspirating the polar body and adjacent MII spindle-containing ooplasm out of the zona pellucida using a 20-μm diameter glass pipette (Humangen, Charlottesville, VA). The enucleation was confirmed by momentary exposure to UV under the control of a shutter system (VCM-D1, Vincent Associates, NY). A donor piPSC that met certain requirements (described in the results) was introduced into the perivitelline space of the enucleated oocyte.

For immediate activation (IA), the oocyte-piPSC couplets were first equilibrated in Fusion Medium (280 mM mannitol solution containing 0.001 mM CaCl2 and 0.05 mM MgSO4) and then transferred to a fusion chamber containing two electrodes overlaid with Activation Medium (260 mM mannitol solution containing 0.1 mM CaCl2 and 0.05 mM MgSO4). Membrane fusion was induced by applying two DC pulses of 160 V/mm for 60 μsec with a cell fusion generator (LF201, Nepa Gene, Chiba, Japan). Then, the oocytes were washed thrice in TLH-BSA and the fusion rates were checked 30 min later. For delayed activation (DA), the oocyte-piPSC cell couplets were first fused with Fusion Medium by two DC pulses of 160 V/mm for 60 μsec. After checking the fusion rate 30 min later, fused ones were incubated in TLH-BSA for another hour and finally activated in Activation Medium by two DC pulses of 120 V/mm for 20 μsec.

### Pseudo-second-polar body extrusion observation, embryo culture and evaluation

After the fusion check (IA), the activated embryos were transferred to PZM-3 in the incubator and the p2PB extrusion rate was checked every 1 h until 5 hpa. For the post-activation 6DMAP treatment experiment, activated embryos were first incubated in PZM-3 supplemented with 2 mM 6DMAP for 3 h. After washing, the embryos were moved to fresh PZM-3 drops. The p2PB extrusion rates were checked at the same time points.

Every 10 reconstructed embryos were cultured in a 30 μl PZM-3 drop at 38.5°C in an atmosphere containing 5% CO2, 5% O2, 90% N2and 100% humidity. Cleavage status was checked at 48 hpa. The numbers of embryos that developed to different pre-implantation stages were counted for each group, and then they were moved to fresh PZM-3 drops. 10% FBS (16000–044, Gibco) was added to each drop at day 4. Blastocyst formation was checked on day 6 of culture. To count the total cell numbers, putative blastocysts were stained with 10 μg/ml Hoechst 33342 for 10 min then mounted and observed under a fluorescent microscope. Total cell numbers were counted according to the pictures taken during observation and only those embryos that possessed more than 30 nuclei were taken into account.

### Immunofluorescent staining of microtubules and microfilaments

To observe the microtubule, microfilament and chromosome dynamics during p2PB extrusion, piPSNT embryos were sampled from control (no 6DMAP) treatment and 6DMAP treatment groups at 0, 1.5, 4 and 8 hpa, respectively. They were fixed with 4% paraformaldehyde for 40 min, then permeabilized with 1% Triton X-100 for 30 min. After incubation in Image-iTTM FX Signal Enhancer (I36933, Invitrogen, Carlsbad, CA) for 30 min, the embryos were further blocked with 1% bovine serum albumin (A9418, Sigma, St. Louis, MO) in PBS for 1 h. They were then incubated with TRITC conjugated phalloidin (1:200, P1951, Sigma, St. Louis, MO) at 37°C for 2 h. After completely washing and secondary blocking, they were further incubated in FITC conjugated monoclonal anti-α-tubulin (1:200, F2168, Sigma, St. Louis, MO) for another 3 h. After the nuclei were stained with 10 μg/ml Hoechst 33342 (B2261, Sigma, St. Louis, MO), the embryos were mounted with anti-fade reagent (S36937, Invitrogen, Carlsbad, CA) and observed under laser-scanning confocal microscope (Zeiss, LSM700).

### Karyotyping analysis

The piPSCs karyotyping analysis was conducted as previously reported [36]. 140 metaphase chromosomal spreads were checked from more than 6 slides. Blastocyst karyotyping analysis was performed according to the previously described protocol by Hao et al. with modifications [37]. Briefly, Day 6 blastocysts were incubated in 0.2 μg/ml Demecolcine (D1925, Sigma) in PZM-3 for 6 h. After the zona pellucida was removed by 5 mg/ml protease (P8811, Sigma), the blastocysts were treated in 0.8% sodium citrate (wt/vol) for 3 min, followed by 0.75 M KCl for 2 min. Then, the blastocysts were fixed in pre-cooled fixing medium (methanol: acetic acid = 3:1 [v/v]) for 30 min. Single blastocysts were transferred to a clean slide and spread with methanol-acetic acid (1:1 [v/v]). After air-drying, the slides were stained with 5% Giemsa and observed under light microscopy with 100x oil immersion objective. 68 blastocysts were subjected to karyotyping from three independent experiments. 62 blastocysts showed at last one metaphase blastomere whose chromosomes were stained and countable. Finally, they were classified by their chromosomal numbers.

### Flow cytometry analysis of cell cycle

Flow cytometry analysis of piPSCs cell cycle was conducted as previously reported with modifications [33]. The piPSCs before and after synchronization were harvested and preplated on uncoated 4-well dishes (176740, Nunc, Denmark) for 30 min to remove the feeder cells. After washing in phosphate buffered saline (PBS) twice, the cells were fixed in pre-cooled 70% ethanol at 4°C overnight. Then, the cells were incubated in staining solution containing 0.1 mg/ml RNase A (27062, iNtRON Biotechnology, Republic of Korea), 50μg/ml propidium iodide (P4170, Sigma) and 0.05% Triton X-100 (X100, Sigma) at 37°C for 40 min. Subsequently, the cells were resuspended in 500 μl PBS. Cell cycle analysis was performed on at least 3 independent cell samples. For each cell population, at least 10,000 cells were analyzed using a FACSCalibur Flow Cytometer (BD Biosciences), and the proportion in G0/G1, S and G2/M phases was estimated using the FlowJo V10 software (Tree Star Inc.).

### Statistical analysis

Each experiment was repeated at least three times. Statistical analysis was conducted using the IBM SPSS Statistics 19 software (IBM Corp., Armonk, NY, USA). A one-way analysis of variance (ANOVA) with least significant difference was conducted to assess the distribution of cell cycle between control and synchronized piPSCs and piPSNT embryo development with different oocyte IVM time; a T-test was conducted to assess the piPSNT embryo development under different activation methods and 6DMAP treatment after pPB extrusion. All data are presented as the mean ± SD. Differences at P < 0.05 were considered statistically significant.

## Results

### Synchronization of porcine induced pluripotent stem cells to metaphase

**Fig 1:**
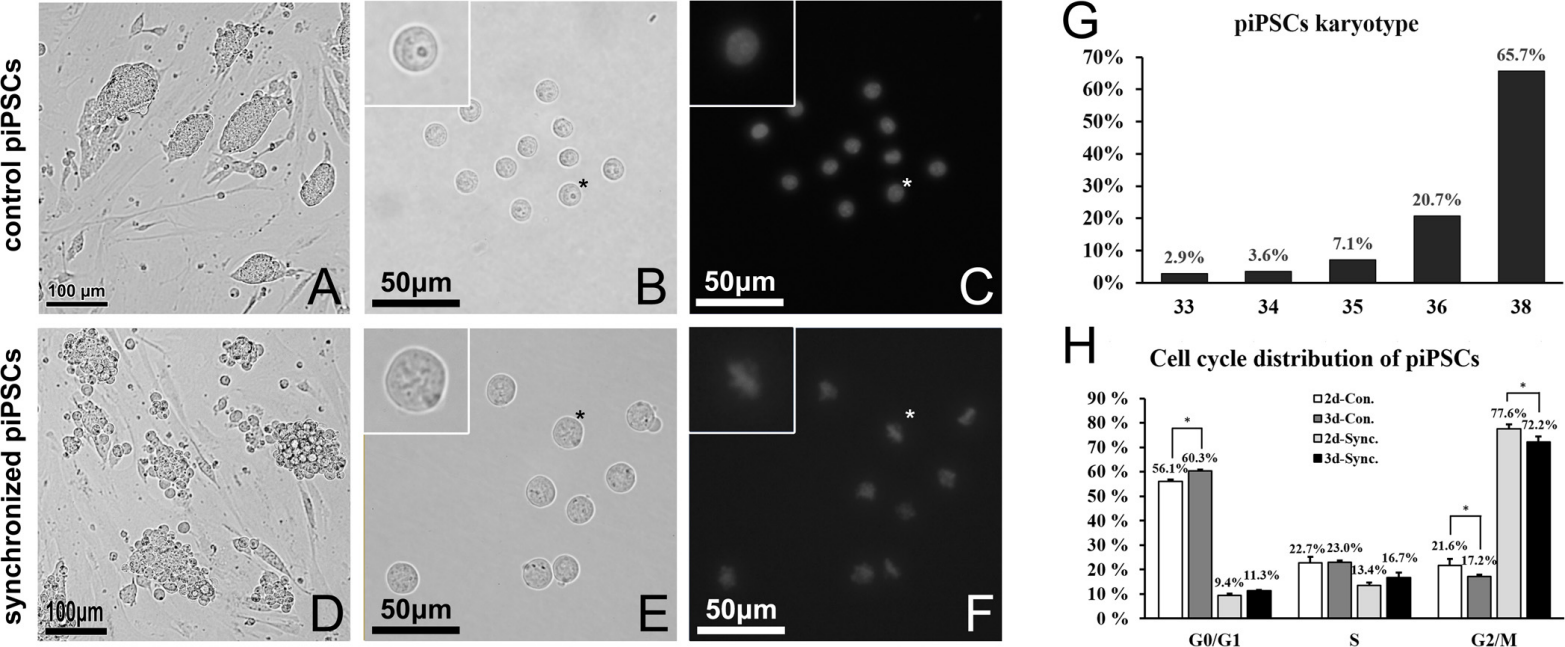
Synchronization of porcine induced pluripotent cells (piPSCs) to metaphase. Control piPSCs showed a typical mouse embryonic stem cell (ESC)-like colony morphology (A). After digestion into single cells by trypsin, small round piPSCs (11–13 μm in diameter) showed high nucleus-to-cytoplasm ratio, with typical G0/G1 nuclei and several nucleoli inside the nuclei (B, C). After the cells were synchronized by a double-blocking method using aphidicolin and nocodazole, round cells emerged on the surface of the piPSCs colony (D) with much bigger cell diameters (17–21 μm) and typical metaphase chromosomal morphology. Some cells also demonstrated linear structures in the middle, which were thought to be the metaphase plate (E, F). The karyotyping analysis was performed using piPSCs at passage 35 (G). 65.7% of cells showed normal karyotype (38 chromosomes) whereas 20.7% of cells had 36 chromosomes. The piPSCs were cultured for 2 or 3 days after subculture and then subjected to synchronization treatment. After harvest, the cell cycle distribution profiles (2d-Sync. and 3d-Sync.) were analyzed by flow cytometry based on PI staining signal and compared with unsynchronized cells (H). After synchronization, most of the piPSCs were arrested at G2/M phase. Moreover, synchronization conducted 2 days after sub-culturing yielded significantly a higher G2/M percentage than 3 days (77.6 ± 1.8% vs. 72.2 ± 2.1%). The details of asterisk marked cells are shown in the upper left corner.

The only known protocol to synchronize piPSCs to metaphase until now is the so-called “double blocking method” [33]. We first tested the effectiveness of this method on our piPSCs line. The synchronization process was conducted on the second or third day after sub-culture. Before cell cycle synchronization, piPSCs colonies had a relative smooth and clear edge (Fig 1A). After being digested into single cells with trypsin, most cells displayed a small round morphology, with their size ranging from 11 to 13 μm (Fig 1B). They had typical G0/G1 or S phase nuclei according to Hoechst 33342 staining (Fig 1C). After synchronization, large round cells emerged within the piPSCs colonies (Fig 1D). After trypsin treatment, most cells had a diameter larger than 17 μm (Fig 1E). A linear structure could be observed in the middle of a large proportion of those cells when they were turned to a certain angle by injection pipette. One hundred cells with diameters ranging from 17 to 19 μm and linear structures were randomly chosen and stained with Hoechst 33342 to check the nuclear status, from which 92 demonstrated a typical mitotic metaphase morphology (Fig 1F). Thus, the same donor cell selection standard was adopted for the following nuclear transfer experiments.

The results of karyotyping using piPSCs at passage 35 showed that 65.7% cells possessed a normal karyotype (38 chromosomes), whereas 20.7% cells had 36 chromosomes due to unknown reasons. The remaining cells had less than 36 chromosomes according to chromosomal spread counting results (Fig 1G). Flow cytometry analysis were conducted to examine the cell cycle distribution with or without synchronization of the piPSCs. The results showed that the cells before synchronization at G0/G1, S and G2/M were 56.1 ± 0.7%, 22.7 ± 2.4% and 21.6 ± 2.7% for day 2 (2d-Con) and 60.3 ± 0.6%, 23.0 ± 0.6% and 17.2 ± 0.7% for day 3 (3d-Con), respectively (S1 Table and Fig 1H). After synchronization, the cells at G0/G1, S and G2/M were 9.4 ± 0.8%, 13.4 ± 1.3% and 77.6 ± 1.8% for day 2 (2d-Sync) and 11.3 ± 0.3%, 16.7 ± 2.0% and 72.2 ± 2.1% for day 3 (3d-Sync), respectively, which means the “double blocking method” also works well in current piPSC lines. Because the 2d-Sync group provided a significantly higher percentage of G2/M cells than the 3d-Sync group, synchronization was conducted on the second day of sub-culture in the following experiments.

### Pseudo-second-polar body extrusion of reconstructed piPSNT embryos

To find a workable protocol for producing piPSNT embryos with a putatively normal karyotype, we first tried to modify our traditional somatic cell nuclear transfer (SCNT), in which 0.4 μg/ml demecolcine and 2 mM 6DMAP were used to treat the reconstructed embryos for 4 h post activation [38]. Because demecolcine is a microtubule-polymerizing inhibitor, which may prevent the p2PB extrusion, we removed it from the post activation treatment.

**Fig 2:**
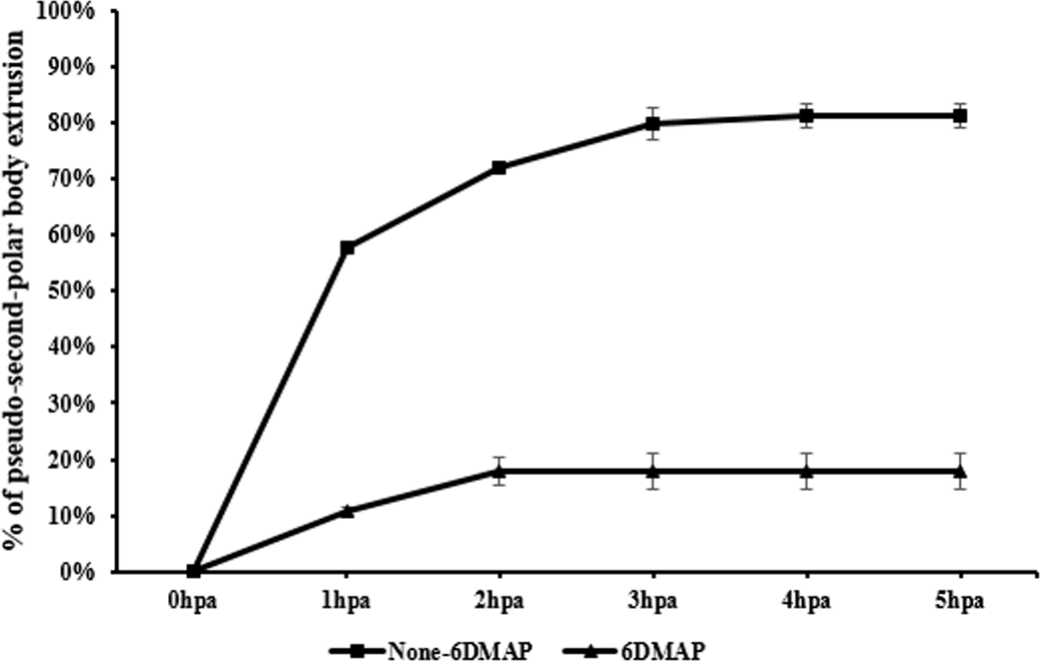
Pseudo-second-polar body (p2PB) extrusion in porcine induced pluripotent cells (piPSCs) embryos post activation. Cloned embryos derived from piPSCs synchronized to metaphase were treated with or without 2 mM 6DMAP for 4 hours post activation (hpa). Embryos that extruded the p2PB were counted every 1 h post activation until 5 hpa. In the 6DMAP treated group, embryos started to extrude p2PBs at 1 hpa and reached the peak at 2 hpa at a rate of 15.8 ± 6.3%. When 6DMAP treatment was removed, 57.6 ± 1.4% of activated embryos extruded p2PBs at 1 hpa. The rate went up to 72.1 ± 2.1% at 2 hpa and reached the peak at 4 hpa of 81.3 ± 4.3%.

After activation, 9.2 ± 1.3% of reconstructed embryos extruded p2PB at 1 hpa. The p2PB extrusion reached a peak at 3 hpa, with a rate of 15.8 ± 6.3% (Fig 2 and S2 Table). We tried removing 6DMAP from the post-activation treatment. Under these conditions, 57.6 ± 1.4% embryos extruded p2PBs at 1hpa. This rate went up to 72.1 ± 2.1 at 2 hpa and reached the peak of 81.3 ± 4.3% at 4hpa.

**Fig 3:**
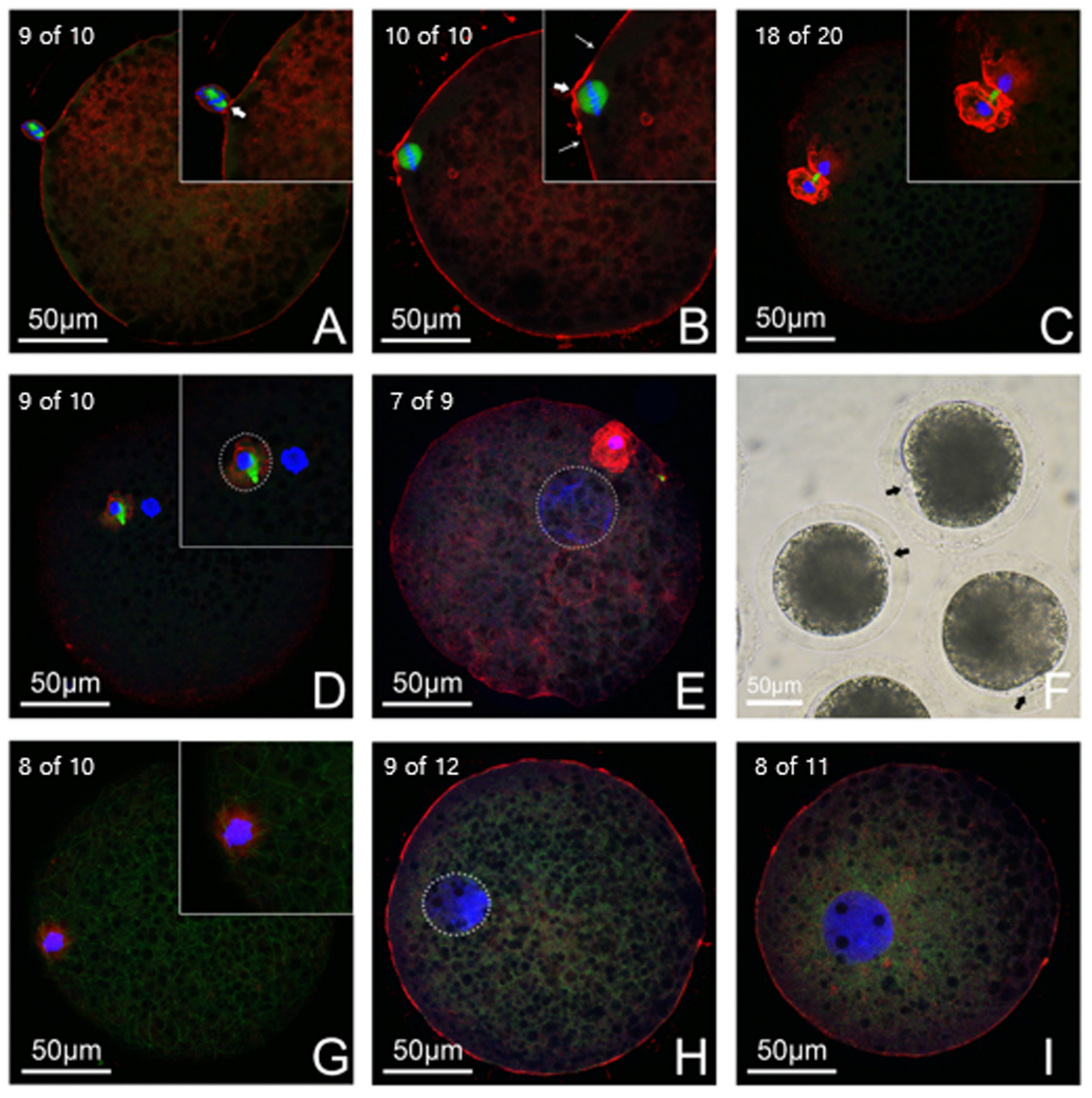
Immuno-fluorescent observation of pseudo-second-polar body (p2PB) extrusion. p2PB extrusion was observed between cloned embryos derived from metaphase piPSCs under 2 mM 6DMAP treatment post activation or not. The microtubules (green), microfilaments (red) and chromosomes (blue) were stained to show p2PB extrusion dynamics. A donor cell-oocyte membrane contact was observed before fusion (A, arrow head). After fusion/activation, a well formed spindle was observed underneath the oocyte membrane with a microfilament cap (B). In the non-6DMAP group, more than half of the embryos extruded p2PB with putative half chromatids at approximately 1.5 hpa (C). A single pronucleus started to form at approximately 4 hpa (D) and it swelled at 8 hpa (E). p2PBs could be easily identified under stereo microscopy (F). In the 6DMAP-treated group, most embryos could not extrude p2PB at 1.5 hpa (G). A single pronucleus formed directly and swelled inside the ooplasm (H, I).

Next we examined the microtubule, microfilament and chromosome dynamics by immuno-fluorescent staining. We found that the membranes of donor cells and oocyte were attached and a metaphase spindle structure was observed within the donor cell before fusion (9 of 10, Fig 3A). After fusion, donor cells were swollen into the ooplasm and a well-formed metaphase spindle emerged underneath the membrane. Moreover, a cortical cone was formed around the spindle (10 of 10, Fig 3B). The non-6DMAP treatment group had a p2PB extruding or extruded out from the reconstructed embryo along with putative half chromatids at 1.5 hpa (18 of 20, Fig 3C). At 4hpa, the p2PB was completely extruded, and the rest of the chromatids started to form a pronucleus (9 of 10, Fig 3D and 3F). At 8hpa, a swelled pronucleus could be observed inside the ooplasm (7 of 9, Fig 3E). In contrast, the 6DMAP treatment group showed that most piPSNT embryos did not extrude p2PBs at 1.5 hpa (8 of 10, Fig 3G). Instead, they directly formed a single pronucleus at 4hpa (9 of 12, Fig 3H), and it swelled at 8 hpa (8 of 11, Fig 3I). Thus, we adopted the former protocol in the following experiments.

### The effect of *in vitro* maturation time on preimplantation development of piPSNT embryos

The synchronization piPSCs to metaphase before nuclear transfer takes long time (15 h in total). To find a convenient and effective time schedule for conducting this study, we examined the effect of different IVM times (40, 44 and 48 h) on further piPSNT embryo development. The results showed that the fused rates, p2PB extrusion rates, 2-cell rates, 4-cell rates and the average cell number of blastocysts had no significant differences among these three groups. However, the 44 and 48 h groups yielded significant more blastocysts than the 40 h group (31.2 ± 2.1% and 33.3 ± 1.7% vs. 25.2 ± 2.0%, P<0.05, Table 1). Thus, 44 h of IVM time was chosen for the following experiments.

**Table 1:** Effect of oocyte in vitro maturation time on piPSNT embryos pre-implantation development.

Group (IVM time)	Reconstructed(Repeat)	Fused	pPB extrusion	No. (%)* of embryos developed to			Total cell number in blastocyst (N)**
				2-cell	≥ 4-cell	blastocyst	
40h	156 (4)	131 (84.0±1.1)	106 (81.0±1.6)	75 (57.3±8.8)	69 (52.7±5.9)	33 (25.2±2.0^a^)	75.3±18.3 (25)
44h	170 (4)	141 (82.9±1.8)	113 (80.1±3.5)	93 (66.0±4.4)	81 (57.5±6.9)	44 (31.2±2.1^b^)	88.9±22.1 (27)
48h	147 (4)	120 (81.8±1.4)	96 (80.0±0.8)	75 (62.5±7.8)	68 (56.7±4.0)	40 (33.3±1.7^b^)	80.9±20.6 (26)

Values with different superscript letters within a column differ significantly (P < 0.05).

*Percentage of fused embryos.

**Number of examined blastocysts.

### The effect of activation method on preimplantation development of piPSNT embryos

When we checked the piPSNT embryos at day 2 for cleavage, more than ten percent of fused embryos demonstrated fragmentation by the above-mentioned protocol (so-called immediate activation, IA). Because the direction at which the donor was fused into enucleated oocyte was random, we speculated that the time between fusion and p2PB extrusion may be too short for some embryos to adjust their “spindle” (from the donor cell) to an appropriate angle before p2PB extrusion, which may result in chromatid segregation errors and subsequent fragmentation. To test this hypothesis, we performed an alternative activation method such that the activation was performed 1 h after fusion (so-called delayed activation, DA) (Table 2). Unfortunately, DA could neither improve the p2PB extrusion rate (77.0 ± 7.3% vs. 79.5 ± 4.0%), 2-cell rate (59.0 ± 12.6% vs. 61.6 ± 5.5%) or 4-cell rate (58.0 ± 15.3% vs. 56.3 ± 5.9%) nor reduce the fragmentation rate (13.0 ± 6.9% vs. 14.3 ± 10.2%) compared to IA. Even worse, it significantly (P<0.05) reduced the fusion and blastocyst formation rate (78.7 ± 6.7% vs. 85.5 ± 5.8% and 16.0 ± 2.1% vs. 31.3 ± 3.3%). Thus, IA was adopted in the following experiments.

**Table 2:** Effect of activation method on piPSNT embryos pre-implantation development.

Group	Reconstructed(Repeat)	Fused	pPB extruded embryos	No. (%)* of embryos developed to			Fragmentation	Total cell number in blastocyst (N)**
				2-cell	≥ 4-cell	blastocyst		
DA	127(4)	100(78.7±6.7 ^a^)	77(77.0±7.3)	59 (59.0±12.6)	58(58.0±15.3)	16(16.0±2.1^a^)	13(13.0±6.9)	69.4±14.7 (13)
IA	131(4)	112(85.5±5.8 ^b^)	89(79.5±4.0)	69 (61.6±5.5)	63(56.3±5.9)	35(31.3±3.3^b^)	16(14.3±10.2)	82.0±21.1 (25)

Values with different superscript letters within a column differ significantly (P < 0.05).

*Percentage of fused embryos.

**Number of examined blastocysts.

### The effect of 6DMAP treatment post activation on preimplantation development of piPSNT embryos

We then examined whether 6DMAP treatment after p2PB extrusion will improve the preimplantation development of piPSNT embryos. The results showed that 6DMAP treatment after p2PB extrusion did not improve the preimplantation developmental competence of piPSNT embryos significantly compared to the untreated group (Table 3), including the 2-cell rate (88.8 ± 4.5% vs. 75.2 ± 14.2%), 4-cell rate (81.3 ± 4.5% vs. 73.3 ± 13.8%) or blastocyst rate (40.2 ± 8.0% vs. 38.1 ± 2.6%, all based on p2PB extruded embryos). The blastocyst average cell number was also not affected (101.9 ± 22.0 vs. 108.0 ± 15.6). Thus, 6DMAP treatment seems to be unnecessary during piPSNT.

**Table 3:** Effect of 6DMAP treatment post pPB extrusion on piPSNT embryos pre-implantation development.

Group	pPB extruded embryos	No. (%)* of embryos developed to			Fragmentation	Total cell number in blastocyst (N)**
		2-cell	≥ 4-cell	blastocyst		
Control	105 (4)	79 (75.2±14.2)	77 (73.3±13.8)	40 (38.1±2.6)	13 (12.4±6.0)	108.0±15.6 (25)
6DMAP	107 (4)	95 (88.8±4.5)	87 (81.3±4.5)	43 (40.2±8.0)	11 (10.3±5.8)	101.9±22.0 (26)

*Percentage of pPB extruted embryos.

**Number of examined blastocysts.

### Characterization of piPSNT embryos

**Fig 4:**
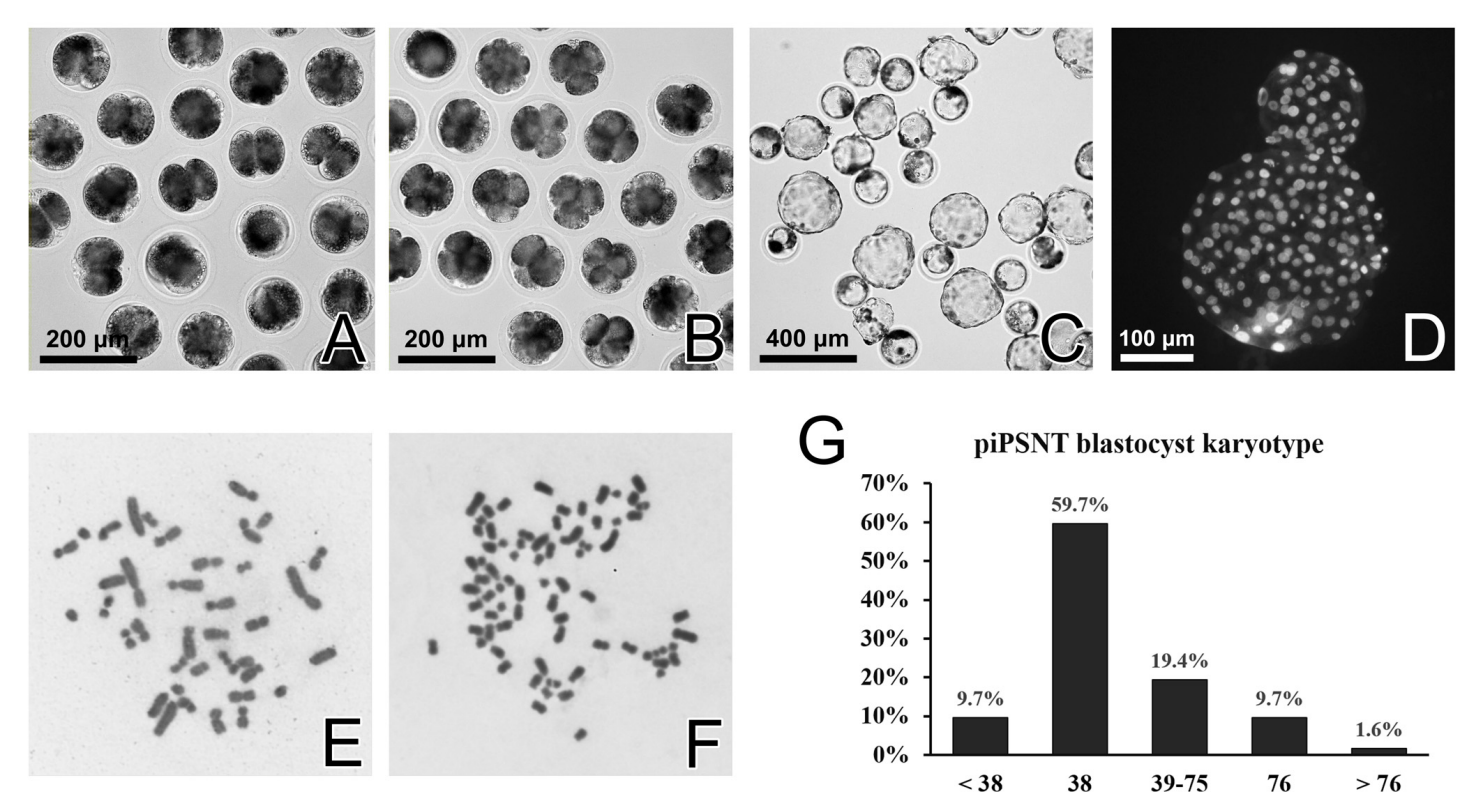
Preimplantation development and characterization of piPSNT embryos. Cloned embryos derived from metaphase piPSCs started to cleave on day 2 (A), developed to 4-cell stage on day 4 (B) and started to form blastocysts on day 6 (C) with good morphology and high total cell numbers (D). The karyotyping results demonstrated that 59.7% of blastocysts possessed normal diploid karyotypes (E, G), while most of the rest were aneuploid (F).

The embryos cloned from metaphase piPSCs with the IA method demonstrated a normal morphology at 2-cell, 4-cell and blastocyst stages (Fig 4A–4D). However, whether p2PB extrusion post-activation would discard half chromatids accurately and leave normal karyotypes in those piPSNT embryos remain unknown. To answer this question, we performed karyotyping on day 6 piPSNT blastocysts. The results showed that 59.7% of blastocysts had normal 2N karyotypes, while the rest of the embryos had either aneuploid or tetraploid karyotypes, indicating that a high proportion of diploid embryos could be obtained by the current method (Fig 4E–4G).

## Discussion

Mouse iPSCs that were synchronized to metaphase by demecolcine treatment were used as donor cells to produce cloned mice and showed a better preimplantation developmental capacity than iPSCs at putative G1 stage and somatic cells [26, 27]. Thus, we speculated that by using piPSCs synchronized to metaphase as donor cells, the preimplanation development capacity of piPSNT embryos could be improved, which may provide new possibilities to produce cloned pigs from piPSCs. Therefore, we investigated the use of piPSCs as donors for efficient production of diploid cloned porcine embryos.

The cell cycle coordination between the recipient cytoplasm of enucleated oocytes and the donor nucleus is critical to prevent chromosomal damage and abnormal ploidy in the resulting embryos [39]. Although pluripotent stem cells such as ESCs and iPSCs are thought to be more efficient donor nuclei during NT due to their self-renewal characteristics and lack of epigenetic memory. They also have a short G1 phase and extended S phase, which are regarded as limited characteristics to act as nuclear donors due to cell cycle coordination. Therefore, in the current study, we adopted a synchronization method called the double-blocking procedure using aphidicolin and nocodazole in a feeder-free culture system as previously reported, in which 94.3% of the cells were successfully arrested to G2/M [33]. Aphidicolin, a reversible inhibitor of eukaryotic nuclear DNA polymerases, blocks the cell cycle at the early S phase [40]. Aphidicolin treatment to PFF after serum deprivation caused the maximal pre-synchronization rates with minimal DNA fragmentation [14]. Nocodazole is a microtubule-depolymerizing drug that blocks cell cycle progression at G2/M [41]. According to previous reports, 70.1% of the cells will enter G1 within 2–3 h after they are released from nocodazole inhibition [33]. In our early trial, nearly 20% of donor cells that were introduced into perivitelline space divided into two daughter cells before fusion). Moreover, mechanical stimulation caused by 16–18 diameter micro-injection pipettes seems to accelerate this process. To reduce the above-mentioned loss, nocodazole, in the same concentration as synchronization medium, was added into all of the media before fusion. In addition, 20 μm micro-injection pipettes were applied and the fusion was conducted immediately when every 30–40 oocyte-piPSC couplets were ready for fusion. Finally, the above mentioned pre-fusion division rate was reduced below 10%. Although piPSCs cultured on MEF feeders were preplated on uncoated tissue culture plates for 30 min to remove the fibroblast feeders before sampling, some fibroblasts were inevitably mixed into the piPSCs samples during flow cytometry analysis. It may explain why we obtained lower a G2/M rate after synchronization. After harvest, however, there were adequate piPSCs that demonstrated typical G2/M morphology under micro-manipulation microscopy, with their diameters ranging from 17–19 μm and a linear structure in the middle, which could be used as donor cells for NT.

To find a workable protocol for piPSNT, we tried to modify the beginning of our conventional SCNT protocol, during which CB and 6DMAP were used post-activation. We firstly removed CB from post-activation treatment because it was a microfilament polymerization inhibitor and would prevent p2PB from extrusion [42, 43]. However, the p2PB extrusion rate was not satisfactorily improved under the conditions of this study. The 6DMAP treatment post-activation has also widely been used in porcine SCNT experiments. Previous studies have shown that it could reduce the intracellular level of maturation promoting factor (MPF) in activated oocyte and keep it low longer [44]. As a protein kinase inhibitor, 6DMAP can inactive the catalytic subunit of MPF, p34cdc2 kinase, by inactivating mitogen-activated protein kinase (MAPK) [45, 46]. Therefore, it has widely been used in mammalian SCNT procedures following electric pulses, to improve the pronuclear formation from donor nucleus and pre-implantation developmental rates of reconstructed embryos [47–49].

However, 0.5 mM 6DMAP treatment for 3 h could make Chinese hamster embryo fibroblasts, CHEF/18 cells, lose their fibroblastic appearance and become round by rapid disruption of the normal structure of microtubules, microfilaments and intermediate filaments [50]. Furthermore, electrically activated porcine oocytes treated with 2 mM 6DMAP for 3 h had a higher incidence of second polar body retention [44]. Therefore, we believe that the low p2PB extrusion rates and high singular pronucleus formation rate of piPSNT embryos in our case were caused by 6DMAP treatment post-activation. Thus, by removing 6DMAP, we achieved a high p2PB extrusion rate, during which half of donor cell chromatids were expelled as confirmed by immuno-fluorescent staining and 2N piPSNT embryos were obtained as confirmed by blastocyst karyotyping. We also attempted to treat those embryos that had already extruded p2PB (checked at 3 hpa) with 2 mM 6DMAP to see whether doing so would affect preimplantation development competence. However, no significant differences were found during cleavage, 4-cell or blastocyst developmental rates, as well as blastocyst cell number compared to control embryos. Because activation with electric pulses alone could decrease MPF and MAPK levels [51], 6DMAP treatment after p2PB extrusion may provide limited benefits towards reducing their levels and is unnecessary for our piPSNT protocol.

After NT, an activation stimulus triggers development of reconstructed embryos. Some reports have shown that delaying oocyte activation after NT can enhance development in cattle and mice [52, 53] through direct exposure of chromosomes to factors in the oocyte cytoplasm before activation, allowing time for the re-assembly of the nuclear apparatus required for the cell cycle. During cleavage check at 48 hpa, we found that 14.3% of embryos demonstrated fragment morphology by the IA method. Because the angle at which the donor cell was fused into oocytes was random but more than half of embryos already extruded p2PB at 1 hpa, we then speculated that the limited time window for p2PB extrusion may cause some reconstructed embryos to suffer from chromatid segregation errors, such as uneven division during p2PB extrusion, which subsequently results in the fragmentation. We then tried DA, in which activation was conducted 1 h later after fusion. Unfortunately, the DA method could neither reduce fragmentation rate, nor improve preimplantation development competence. To the contrary, it significantly reduced the fusion and blastocyst rates. The two instances of electric stimulation may increase the possibility of intracytoplasmic disorder or damage and could be responsible for the results described above.

We anticipated that piPSNT embryos would lack the epigenetic memory of somatic cell types. Therefore, they might be more readily reprogrammed within the ooplasm after NT and a methodology using piPSCs as donors could result in homozygous NT embryos in just one generation and more efficient cloned offspring with fewer abnormalities. However, according to our previous report, porcine ESCs derived from piPSNT blastocysts (iPS-NT ESCs) showed more naïve like features in gene expression and immature mitochondrial morphology but aberrant patterns in the inactivation of X chromosomes despite having normal karyotypes distinguished them from those derived from other origins, including IVF and parthenogenetic activation [54]. Therefore, more experiments are warranted to explore the epigenetic status of the final piPSNT embryos and additional studies regarding its role in producing viable cloned piglets are needed.

Nevertheless, it is worth mentioning here that the modified protocol in this study enhanced the rate of reconstructed embryos with normal ploidy using G2/M- stage iPSCs as donors (59.7%) compared to that of the NT embryos in previous reports (25%) [33]. Additionally, this enhanced diploid rate is much higher than that of NT embryos using G2/M stage fetal fibroblasts (37.9%) [17]. The prerequisites of using metaphase piPSCs as donor cells for porcine NT are that they can be efficiently synchronized to metaphase and half of their mitotic chromatids could be successfully extruded out of ooplasm post-activation, leaving embryos with normal karyotypes. However, porcine MII oocytes are thought to be less efficient than mouse oocytes in permitting the extrusion of extra chromosomes derived from G2/M stage donors called pPB. A much lower frequency of aneuploidy in this study might be associated with the improvement of the pPB extrusion. This finding was in agreement with the results obtained by the immunofluorescence assay showing the dynamics of the p2PB extrusion via staining of microtubules and microfilaments. This result suggests that this protocol has an ability to retain a stable diploid complement of chromosomes in embryos reconstituted from iPSCs, thus leading to success towards generating cloned pups from porcine iPSCs.

In summary, by using synchronized metaphase piPSCs as donor cells and enucleated MII oocytes as recipients, and coupled with the IA method, we could stably produce piPSNT blastocysts with normal morphology and karyotypes. In conclusion, the current study developed a state-of-the art protocol for producing piPSNT embryos, which were derived from piPSCs at G2/M but possessed normal karyotype. From a broad perspective, it provides a valuable reference for future study on stem-like cell nuclear transfer experiments in pig, as well as in other domestic animals.

## Supporting Information

S1 Table Distribution of different cell cycle phased of porcine induced pluripotent cells with or without synchronization. (DOCX)

S2 Table Pseudo-second-polar body extrusion of porcine induced pluripotent cell nuclear transfer embryos reconstructed from metaphase donor cells. (DOCX)
