# Supplementary material for: An Improved System for Generation of Diploid Cloned Porcine Embryos Using Induced Pluripotent Stem Cells Synchronized to Metaphase
Source: PLoS One. 2016 Jul 29;11(7):e0160289. doi: 10.1371/journal.pone.0160289 (PMC4966966; doi:10.1371/journal.pone.0160289)
Supplement: S1 Table — (DOCX) [file pone.0160289.s001.docx]

**S1 Table.** **Distribution of different cell cycle phased of porcine induced pluripotent cells with or without synchronization**.

| Group | G_0_/G_1_(%) | S(%) | G_2_/M(%) |
| --- | --- | --- | --- |
| 2d-Con. | 56.1±0.7^a^ | 22.7±2.4^a^ | 21.6±2.7^a^ |
| 3d-Con. | 60.3±0.6^b^ | 23.0±0.6^a^ | 17.2±0.7^b^ |
| 2d-Sync. | 9.4±0.8^c^ | 13.4±1.3^b^ | 77.6±1.8^c^ |
| 3d-Sync. | 11.3±0.3^d^ | 16.7±2.0^c^ | 72.2±2.1^d^ |

_The data were presented as Mean ± SD. Values with different superscript letters within a column differ significantly (P < 0.05)._
