# Supplementary material for: An Improved System for Generation of Diploid Cloned Porcine Embryos Using Induced Pluripotent Stem Cells Synchronized to Metaphase
Source: PLoS One. 2016 Jul 29;11(7):e0160289. doi: 10.1371/journal.pone.0160289 (PMC4966966; doi:10.1371/journal.pone.0160289)
Supplement: S2 Table — (DOCX) [file pone.0160289.s002.docx]

**S2 Table. Pseudo-second-polar body extrusion of porcine induced pluripotent cell nuclear transfer embryos reconstructed from metaphase donor cells.**

| Group | 1hpa (%) | 2hpa (%) | 3hpa (%) | 4hpa (%) | 5hpa (%) |
| --- | --- | --- | --- | --- | --- |
| None-6DMAP | 57.6±1.4^a^ | 72.1±2.1^a^ | 79.6±5.7^a^ | 81.3±4.3^a^ | 81.3±4.3^a^ |
| 6DMAP | 9.2±1.3^b^ | 14.4±5.0^b^ | 15.8±6.3^b^ | 15.8±6.3^b^ | 15.8±6.3^b^ |

_The data were presented as Mean± SD. Values with different superscript letters within a column differ significantly (P < 0.05_
